# GBWOEM: A Gradient-Based Weight Optimization Model for Improved Predictive Accuracy in Healthcare

**DOI:** 10.12688/f1000research.169436.2

**Published:** 2026-01-27

**Authors:** Surajit Das, Samaleswari P. Nayak, Biswajit Sahoo, Satyananda Champati Rai

**Affiliations:** 1 School of Computer Engineering, Kalinga Institute of Industrial Technology, Bhubaneswar, Odisha, 751024, India; 2 Department of Computer Science and Engineering, Silicon University, Bhubaneswar, Odisha, 751024, India

**Keywords:** Ensemble Learning, Healthcare, Weight Optimization, GBWOEM, Classification, ROC Curve, Machine Learning, Predictive Accuracy, AUC.

## Abstract

**Background:**

The use of ensemble learning has been crucial for improving predictive accuracy in healthcare, especially with regard to critical diagnostic and classification problems. Ensemble models combine the strengths of multiple ML models and reduce the risk of misclassification, which is important in healthcare, where accurate predictions impact patient outcomes.

**Methods:**

This study introduces the Gradient-Based Weight Optimized Ensemble Model (GBWOEM), an advanced ensemble technique that optimizes the weights of five base models: Decision Tree Classifier (DTC), Random Forest Classifier (RFC), Logistic Regression (LR), Multi-Layer Perceptron (MLP), and K-Nearest Neighbours (KNN), through optimizing the weights. Two variants, GBWOEM-R (random weight initialization) and GBWOEM-U (uniform weight initialization), were proposed and tested on five healthcare-related datasets: breast cancer, Pima Indians Diabetes Database, diabetic retinopathy debrecen, obesity level estimation based on physical condition and eating habits, and thyroid diseases.

**Results:**

The test accuracy of the proposed models increased to 0.48-8.26% over the traditional ensemble models, such as Adaboost, Catboost, GradientBoost, LightGBM, and XGBoost. Performance metrics, including ROC-AUC analyses, confirmed the model’s efficacy in handling imbalanced data, highlighting its potential for advancing predictive consistency in healthcare applications.

**Conclusion:**

The GBWOEM model improves the predictive accuracy and offers a reliable solution for healthcare applications even when dealing with the imbalance data. This strategy has the potential to ensure patient outcomes and diagnostic consistency in healthcare settings.

## Introduction

In recent times, the use of ML in healthcare has gained enormous popularity owing to the increasing need for high-quality, timely, and efficient predictions, which are necessary for reliable diagnosis as well as assisting patients and physicians in treatment planning. Given that healthcare is intrinsically steeped in complex, high-stakes decision-making processes, this prediction errors can be catastrophic to patient care. This has led to the need to develop machine learning models that are not only accurate in prediction, but also transparent, generalizable across broad patient populations and practice settings, and robust against small errors in input features. Ensemble learning is a powerful approach among ML methodologies. Ensemble learning improves the overall performance of the system by combining multiple base models to create a single model that strengthens each other while covering their individual. ^1^ This model aggregation decreases the variance and reduces bias and overfitting. This is particularly important when using healthcare datasets because they are often complex, that is, high-dimensional, noisy, imbalanced, or even insufficient. Therefore, there is an abundance of healthcare applications where ensemble learning has been applied successfully, such as disease prediction, medical image analysis, and patient outcome forecasting. ^2, 3^

Recently, ensemble methods, such as Bagging, ^4^ Boosting ^5^ and Stacking, ^6^ have been the focus of many predictive systems because they promise state-of-the-art performance in many domains. The most obvious bagging technique is the Random Forest, which boosts the stability and accuracy of models by training many weak learners independently and then combining their predictions. Boosting algorithms such as Adaboost, GradientBoost, XGBoost, and Catboost adjust the weights for the current model in some ways and continue throughout the predictions to correct any errors, which improves the accuracy of the overall ensemble. Instead, stacking trains is a metamodel that uses predictions from various base models, which usually results in more sophisticated predictions. When applied in healthcare, they have shown important results, allowing researchers and clinicians to develop models that can more accurately predict disease onset, severity, and treatment effectiveness.

However, there is scope for improvement in tuning the contribution of each base model to the ensemble. Traditional approaches tend to treat all base models equally or correct model weights using ad-hoc rules. The shrugging of singular complexities existing in healthcare data has, understandably, failed for most. In healthcare applications, an imbalanced dataset is a common issue where the data only have a few positive classes, and if not handled properly, one will come out with a model that has bias. Furthermore, the heterogeneity of healthcare datasets, where feature spaces and data distributions vary widely, presents a significant challenge for traditional ensemble methods. ^7, 8^

To address these challenges, we propose a novel Gradient-Based Weight Optimized Ensemble Model (GBWOEM), which is a self-adjusting ensemble learning model in which the weights of individual base models are dynamically assigned and updated via gradient-based optimization. This method assigns more weight to models that make better predictions and reduces the impact of weaker models to improve the overall performance of the ensemble. The sensitivity of the model is extremely high, which enables the minority class to be predicted more accurately. In our empirical evaluation, two major variants of the model were considered, GBWOEM-R and GBWOEM-U, to study and present a performance comparison with existing models. The key contributions of this study are as follows:

• Developed the Gradient-Based Weight Optimized Ensemble Model (GBWOEM) with two different weight initialization strategies (GBWOEM-R and GBWOEM-U), a novel way to optimize the base model’s weights in an ensemble dynamically.
• Introduced a log-based loss function with a small constant ε to ensure numerical stability and refine the model’s performance on imbalanced datasets.
• Experiments were conducted on five diverse datasets from the healthcare field, showing that the proposed GBWOEM consistently outperforms popular ensemble models such as Adaboost, Catboost, XGBoost, LightGBM and Gradient Boosting across all cases.
• Proved to is robust and adaptable to varying data contexts that show improvements in test accuracy for each of the datasets, ranging from 0.48% to 8.26%.
• We evaluated the relative weights assigned to individual base models in ensemble combinations, giving us useful information about what each contributing base model was doing with respect to the final prediction.

## Related work

Several studies have demonstrated that ensemble models are effective in a range of medical classification tasks. In a study by Younas et al. ^8^ used a weighted average ensemble technique combining GoogleNet and ResNet-50 to classify colorectal polyps using an augmented dataset (Gastrointestinal Lesions in Regular Colonoscopy and PICCOLO). Their ensemble model outperformed the base models and some other CNN-based deep neural networks, such as Inception-v3, Xception, DenseNet-20, and SqueezeNet. Bhuiyan and Islam ^9^ used weighted average and maximum voting ensemble techniques to ensemble VGG16, VGG19, and DenseNet201 for malaria classification from red blood cell images. The authors achieved improved performance over weight-based ensemble models and different CNN-ML classifiers. Marques et al. ^10^ proposed a cross-validation-based ensemble model using EfficientNetB0, averaging predictions across folds, for malaria detection, and achieved superior results compared to other researchers. Ali et al. ^11^ proposed a bagging-based ensemble model using a DNN to predict problems in the heart. The results of different networks are combined using Logit Boost, which achieves a better performance than Support Vector Machine (SVM), Logistic Regression (LR), Multi-Layer Perceptron (MLP), Random Forest Classifier (RFC), etc. Dutta et al. ^12^ introduced a weighted average-based ensemble with models such as Gaussian Naïve Base (GNB), Decision Tree (DT), XGBoost (XGB), Random Forest (RF), and LightGBM (LGB) for early diabetes prediction, although with limited accuracy.

The boosting-based ensemble model id proposed by Ihnaini et al. ^13^ to predict diabetes, where trees were used as weak learners, outperforming LR, NB, RF, K-Nearest Neighbour (KNN), DT, and SVM. Reddy et al. ^14^ used a voting-based ensemble model by combining LR, KNN, RF, DT, and AdaBoost with voting for diabetic retinopathy classification, which outperformed the base models. Habib and Tasnim ^15^ used the hard voting technique to form an ensemble model by combining LR, NB, RF, and MLP to classify cardiovascular diseases and outperformed the base models. For brain tumor classification, Al Amin et al. ^16^ used majority voting over ResNet-50, DenseNet121, InceptionV3, VGG19, and VGG16 for brain tumor classification, achieving a higher validation accuracy. El-Sappagh et al. ^17^ used different ensemble strategies such as majority voting, weighted majority voting, and stacking on the top of different base models such as SVM, MLP, RF, DT, KNN, LR, and XGB for Alzheimer’s disease classification, concluding that stacking with XGBoost is most effective. De Souza et al. ^18^ applied stacking with CNN, LSTM, and CNN-LSTM to anxiety classification. For tuberculosis classification, Osamor and Okezie ^19^ used a weighted voting ensemble with NB and SVM with PCA and RFE-CV feature selection, achieving notable accuracy despite its simplicity.

Using a federated learning-based setup, Subashchandrabose et al. ^20^ proposed a decentralized ensemble model for lung cancer classification and compared its performance with other ML models in both centralized and decentralized architectures, concluding that the proposed model works better in decentralized architecture. Abbas et al. ^21^ improved lung cancer classification using a weighted federated ensemble, optimizing the weight of the client’s ANN using the Levenberg− Marquardt and Bayesian regularization techniques, and their weighted sum was used in the server model for the final classification. Kotei and Thirunavukarasu ^22^ proposed a stacking-based ensemble of nine pre-trained CNN for tuberculosis classification. Despite its high accuracy, the model size and resource utilization are significant. Regarding diabetes classification, Prakash et al. ^23^ used hard voting with an ANN, RNN, DBN, Perceptron, and RDF, finding it better than Bagging, Boosting, and Stacking. EL-Rashidy et al. ^24^ proposed a stacking-based ensemble for the classification of mortality using KNN, MLP, LDA, DT, and LR, and stacked them using LR as a meta-learner, showing better results for other ensembles.

For Covid-19 classification based on Chest X-ray images, Rajaraman et al. ^25^ proposed an ensemble model using CNN and ImageNet and found that the weighted average is the most efficient. For TB classification Rajaraman and Antani ^26^ proposed a CNN-based stacking ensemble using pre-trained models, such as Inception-v3, CNN, VGG-16, InceptionReseNet-V2, Xception, and Densenet-121. The model achieved good accuracy at the cost of an increased model size. Juraev et al. ^27^ accessed different static and dynamic ensemble strategies and concluded that the DESKNN strategy yielded the best results when classic ML models were used as the base models. Anand et al. ^28^ used a weighted average-based ensemble for the classification of brain tumors using VGG19 and variants of CNN, initializing weights using grid search and achieving better performance than base models.

Through the survey shown in Table 1, a significant difference in the usage of base models based on the type of data was observed. Researchers have mostly used statistical or other traditional models as base models when working with tabular data, where the data are arranged in rows and columns, such as medical records or diagnostic metrics, whereas CNNs are typically employed as base models for image datasets. The use of CNN-based models results in an increase in the complexity and computational footprint compared with other ensemble models. A range of ensemble techniques, such as averaging, aggregation, weighted averaging, voting, weighted voting, boosting, and bagging, have been used to integrate the results of diverse base models. Of all the mentioned techniques, weighted averaging is one of the most commonly used techniques for combining results owing to its effectiveness in improving ensemble performance.

**Table 1.** Literature review.

Author	Disease	Models	Ensemble technique	Accuracy	Observation
Dutta et al. ^12^	Diabetes	GNB, BNB, RF, DT, XGB, LGB	Weighted Average	73.5	The AUC of the base models is taken as weight.
Amin et al. ^16^	Brain Tumor	ResNet-50, DenseNet121, InceptionV3, VGG19, VGG16	Voting	98	Proposed model outperforms base models.
Habib and Tasnim ^15^	Cardio vascular Disease	LR, GNB, RF, MLP	Voting	88.42	Proposed model outperforms base models.
Baha et al. ^13^	Diabetes	Trees	Boosting	99.6	Proposed ensemble outperforms existing ML models.
Kotei and Thirunavukarasu ^22^	Tuber-culosis	VGG16, VGG19, InceptionV2, MobileNet, Xception, Densenet, EfficientNEtB1, Resnet50, InceptionV3, CNN	Stacking	98.38	Too many CNN-based models increase the complexity and computational footprint.
Younas et al. ^8^	Colorectal Cancer	GoogleNet, ResNet-50	Weighted Average	96.3	Grid search is used for weight initialization.
Ali et al. ^11^	Heart Disease	DNN, LogitBoost	Weighted Average	98.5	All the experimental models gave their respective highest accuracy at the same feature count.
Juraev et al. ^27^	Mortality	DT, LR, Linear SVR, KNN, Lidge, Lasso, CB, XGB, RF, GB, LGBM	Voting	98.7	Traditional ensemble models as base model reduces the model gives better performance.
Reddy et al. ^14^	Diabetic Retinopathy	LR, DT, KNN, RF, Adaboost	Voting	82	Proposed model outperforms base models.
Marques et al. ^10^	Malaria	EfficientNetB0	Averaging	98.29	Average of 10-fold cross-validation is used.
Bhuiyan and Islam ^9^	Malaria	VGG16, VGG19, DenseNet201	Weighted Average, Max Voting	97.92	Proposed model outperforms base models.
EL-Rashidy et al. ^24^	Mortality	KNN, MLP, LDA, DT, LR	Stacking	94.4	Performs better than existing ensembles.
Prakash et al. ^23^	Diabetes	ANN, RNN, DBN, Perceptron, RDF	Voting	92	Voting is giving better results than the other ensemble techniques.
Abbas et al. ^21^	Lung Cancer	ANN	Weighted Sum	96.3	Model works better only in distributed system.
Shaker et al. ^17^	Alzheimer’s disease	SVM, MLP, RF, DT, KNN, LR, XGB	Majority voting, Weighted majority voting, stacking	89.15	Stacking with XGB performs better than the other setups.
Rajaraman and Antani ^26^	Tuber-culosis	Xception, Densenet-121, CNN, VGG-16, Inception-v3, InceptionReseNet-V2	Stacking	94.1	So many pre-trained models increase the computational footprint.
Rajaraman et al. ^25^	Covid-19	CNN, ImageNet	Weighted Average	99.01	Weighted average with pruned CNN model performs better.
Subashchandrabose et al. ^20^	Lung Cancer	NN	-	89.63	The centralized approach gave a better result than the decentralized approach.
Souza et al. ^18^	Anxiety	CNN, LSTM	Stacking		A blend of CNN, LSTM, and CNN-LSTM with stacking gave less error than others.
Anand et al. ^28^	Brain Tumor	VGG19, CNN	Weighted Average	98	Grid search is used for weight initialization.
Osamor and Okezie ^19^	Tuber-culosis	NB, SVM	Weighted Voting	96	Simple ensemble model with better performance.

## Proposed methodology

**Figure 1.**
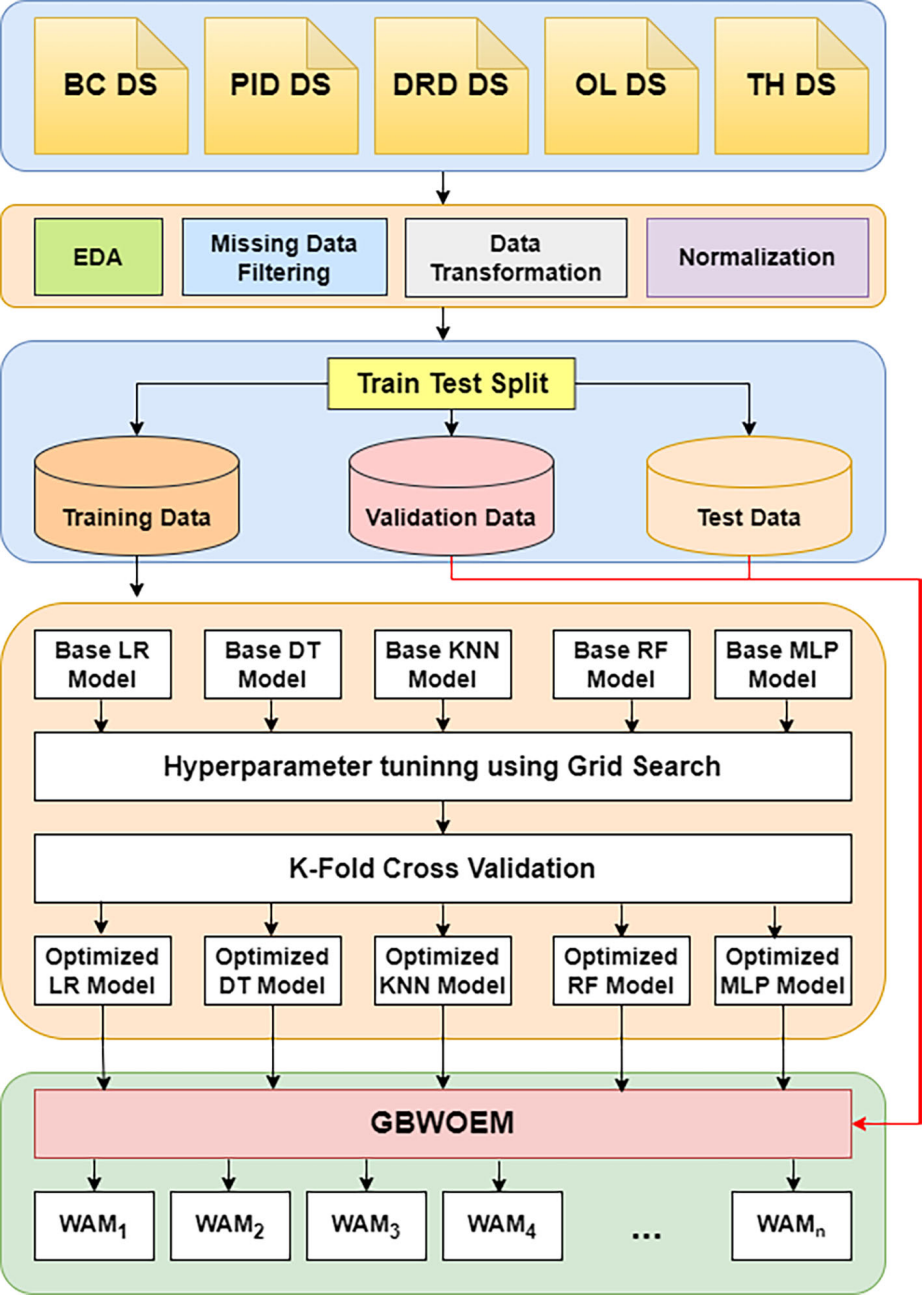
Proposed methodology.

A new Gradient-Based Weight Optimized Ensemble Model (GBWOEM), which uses a variety of base models to improve prediction performance, has been introduced. Figure 1 provides a full overview of the GBWOEM’s design. A dataset is first obtained from the UCI repository, and then extensive Exploratory Data Analysis (EDA), pre-processing, data transformation, and normalization are performed to ensure that the data are ready for modelling. To enable extensive model evaluation and performance assessment, the dataset was divided into training, validation, and testing sets. We chose five diverse base models for the ensemble model, each representing a different modelling approach: Logistic Regression (LR) for statistical strength, Decision Tree Classifier (DTC) for interpretability, Random Forest Classifier (RFC) for robustness and ensemble capabilities, Multi-Layer Perceptron (MLP) for deep learning, and K-Nearest Neighbours (KNN) for distance-based methodology.

Grid Search is used to improve hyperparameters for each of these foundation models, and K-Fold Cross-Validation is used to guarantee generalization across various training data subsets. Five separately optimized models are the end product of this method. The GBWOEM iteratively selects combinations of base models ( ${5_{C}}_{2},{5_{C}}_{3},{5_{C}}_{4},\text{and}\;{5_{C}}_{5}$ ) or Weighted Average Models (WAM) using the five optimized models that are input. Using the validation dataset, the GBWOEM adjusts the weights of the chosen base models during each iteration to ascertain their respective contributions to the ensemble. The effectiveness of the ensemble is then determined by evaluating its performance on a test dataset. The combinatorial possibilities of choosing various subsets of the five base models yields 26 potential combinations because of the GBWOEM method. By assessing the performance of every combination on the test set, GBWOEM determines the ensemble configuration that enhances the accuracy of the predictions. With a combination of statistical, distance-based, tree-based, and neural network techniques, the final model is chosen based on best predictive performance out of all the basic models.

### Datasets

While developing a model, it is important to evaluate its performance on a variety of datasets. Different datasets represent different disease domains such as diabetes, cancer, thyroid, and obesity. Each dataset has distinct properties, including differences in feature types, noise level, and distribution. Testing the model’s performance across these broad domains helps determine the model’s ability to generalize new data. Using different datasets with diverse qualities and dimensions, evaluate the model’s robustness and determine whether the model consistently performs well or is impacted by the nature of the data. It can identify areas where the model can be improved, such as by handling imbalanced data or dealing with noise. The analysis of performance inconsistencies can aid in model adjustment and fine-tuning. Information regarding the datasets used in this experiment is presented in Table 2.

**Table 2.** Information of the dataset.

Name of the datasets	Number of instances	Number of features	Target variable	Data distribution	Missing value present?	Feature type
Breast Cancer Dataset (BC DS) ^29^	569	32	Diagnosis Malignant/M = 1 Benign/B = 0	1: 37.3% 0: 62.7%	No	Numerical
Diabetic Retinopathy Debrecen (DRD DS) ^30^	1151	20	Diabetic Retinopathy (1, 0)	1: 52.9% 0: 47.1%	No	Numerical
Pima Indians Diabetes Database (PID DS) ^31^	768	9	Outcome (1, 0)	1: 34.9% 0: 65.1%	Yes	Numerical
Obesity Dataset (OL DS) ^32^	2111	17	Obesity Level Normal (0), Obesity (1)	1: 46.6% 0: 53.4%	No	Numerical, Categorical
Thyroid Disease (TH DS) ^33^	3772	30	Thyroid Disease (1, 0)	1: 91.8% 0: 8.2%	Yes	Numerical, Categorical

### Base models

An ensemble model in machine learning makes predictions by combining several “base learners” or “base estimators”, each of which performs a classification or prediction task. In our proposed work, to build the GBWOEM, Decision Tree Classifier (DTC), Logistic Regression (LR), Random Forest Classifier (RFC), K-Nearest Neighbour (KNN), and Multi-Layer Perceptron (MLP) is used.

LR is a widely used empirical model in clinical analyses. It serves multiple purposes, including classification and feature selection, and as a meta-learner in ensemble models. ^34– 36^ As a supervised ML algorithm, binary classification is the primary application of LR. It evaluates the relationship between one or more independent variables and categorizes data into distinct classes. Decision trees are used in complex decision-making processes or to predict patient outcomes based on features from large datasets. ^37– 39^ The trees divide data on the basis of feature values and provide optimal decisions with respect to certain criteria, for example, Gini impurity $G=1-\sum_{i=1}^{n}p_{i}^{2}$ or information gain IG=H(Y)−H(Y|X) , where H is entropy and p _i_ is the probability of class. A data point is assigned with a label of the majority class among its k nearest neighbours by the k-NN algorithm, where k is a user-specified hyperparameter. It matches these neighbours using distance metrics, such as the Euclidean distance $d=\sqrt{\sum_{i=1}^{n}{\left(x_{i}-y_{i}\right)}^{2}}.$ ^40^ Methods such as bagging and boosting can be utilized with k-NN to develop more robust models that reduce noise sensitivity and improve the accuracy. ^41^ Random Forests combines multiple decision trees trained on bootstrap samples with a random subset of features, ^42^ significantly reducing overfitting. ^43^ Taking this into consideration in the field of healthcare, it can be utilized with tasks pertaining to disease classification, risk prediction, and patient outcome forecasting. MLPs are deep learning models capable of performing complicated classification and regression tasks because their ability is to represent complex non-linear relationships using multiple layers of neurons. ^44^ Using backpropagation and gradient descent, it minimizes a loss function and subsequently the network learns, where the loss function for classification tasks might be cross-entropy $L=-\sum_{i=1}^{n}\;y_{i}\log\left(\hat{y_{i}}\right)$ and for regression tasks, it might be MSE. The activation functions used in the hidden layers, such as ReLU or sigmoid MLP, introduce non-linearity, ^45^ allowing them to pick up complex patterns in the data.

LR, DT, kNN, RF, and MLP were considered for our ensemble model. While LR models can only capture linear relationships, DTs describe non-linear interactions and variable importance. Although RFs improve ensemble performance by combining many decision trees, they are designed to promote generalization over a wide range of datasets. MLPs can capture highly non-linear relationships in large datasets with relatively complex feature representations. The KNN learns the local structure and variance, leading to fine-grained predictions based on data closeness.

### GBWOEM

**Figure 2.**
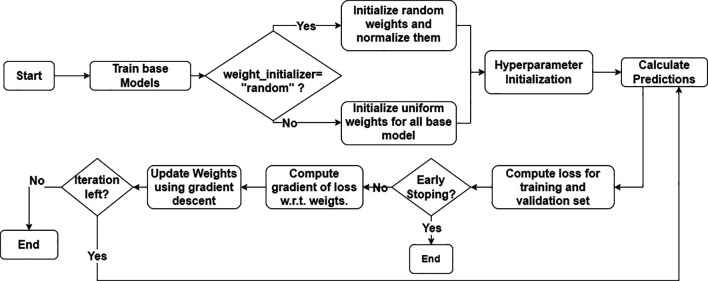
Process flow of GBOWEM.

The Gradient-Based Weight Optimized Ensemble Model (GBWOEM) is a weighted average-based ensemble model that integrates five diverse base models: LR, DT, KNN, RF, and MLP. Figure 2 shows a flowchart of the algorithm. The ensemble approach harnesses the strengths of each base model, and the final prediction is calculated by averaging the outputs with appropriate weights. GBWOEM assigns and updates weights to each base model in a systematic manner based on the performance of the base models. The entire process begins by training the base models. Every base model is trained rigorously using a grid search CV along with k-folds to extract the best model out of all variations. The model with the best accuracy among the trained models is selected as the initial candidate that will undergo optimization through the GBWOEM algorithm.

The first step in weight optimization is to establish the weights for every base model using two modes, GBWOEM-R, where the weights are randomly initialized and normalized to keep their sum equal to one. Alternatively, in GBWOEM-U, the weights are initialized uniformly. After the weight assignment, other hyper-parameters, including learning rate, number of iterations, and a patience parameter to stop early, are initialized. Subsequently, with these initial weights, the ensemble provides its first predictions based on the initial results and evaluates the ensemble using a loss function, which includes both training and validation losses. When the model fails to meet an early stopping condition, that is, if the validation loss does not improve within a certain number of iterations, the training is terminated so that overfitting can be avoided. However, if the stopping condition is not satisfied, the next phase involves calculating the gradient of the loss function with respect to the weights of the base models. Subsequently, the weights are updated using a gradient descent optimization technique to reduce the overall ensemble loss. This process is continued until the maximum number of iterations is reached, or the early stopping condition is met. The process is described in Algorithm 1.

Algorithm 1. **Gradient Based Weight Optimized Ensemble Model (GBWOEM).**

**Table:** 

**Input:**	- Learning rate ( *α*) - Number of iterations ( *num iterations*) - Patience for early stopping ( *patience*) - Weight initializer method ( *weight initializer*)
**Output:**	- Trained ensemble model with optimized weights
**Parameters:**	- Number of base models: n - Base models: $\left\{M_{1},M_{2},\ldots ,M_{n}\right\}$ - Ensemble predictions on training set: $\left\{{\hat{Y}}_{\text{train}}^{\left(1\right)},{\hat{Y}}_{\text{train}}^{\left(2\right)},\ldots ,{\hat{Y}}_{\text{train}}^{\left(n\right)}\right\}$ - Ensemble predictions on validation set: $\left\{{\hat{Y}}_{\mathrm{val}}^{\left(1\right)},{\hat{Y}}_{\mathrm{val}}^{\left(2\right)},\ldots ,{\hat{Y}}_{\mathrm{val}}^{\left(n\right)}\right\}$ - Ensemble predictions on test set: $\left\{{\hat{Y}}_{\text{test}}^{\left(1\right)},{\hat{Y}}_{\text{test}}^{\left(2\right)},\ldots ,{\hat{Y}}_{\text{test}}^{\left(n\right)}\right\}$ - Weights: $W\left\{w_{1},w_{2},\ldots ,w_{n}\right\}$
1. If weight_initializer == ‘random’: 2. W = [n random number sampled uniformly from [0,1)] 3. $W_{\text{normalized}}$ = $\frac{W}{\sum W}$ 4. W = $W_{\text{normalized}}$ 5. Else If weight_initializer == ‘uniform’: 6. W = ones_array/n 7. Else: 8. Value Error 9. For each base model $M_{i}:$ 10. Fit $M_{i}$ on training data ( $X_{\text{train}},Y_{\text{train}}$ ) 11. Predict probabilities for $X_{\text{train}},X_{\mathrm{val}},X_{\text{test}}$ 12. Store Predictions ${\hat{Y}}_{\text{train}}^{\left(1\right)},{\hat{Y}}_{\text{train}}^{\left(2\right)},\ldots ,{\hat{Y}}_{\text{train}}^{\left(n\right)}$ 13. For iteration t in range (num_iterations): 14. Compute ensemble predictions using current weights: 15. ${\hat{Y}}_{\text{ensemble},\text{train}}^{\left(t\right)}=\sum_{i=1}^{n}w_{i}^{\left(t\right)}.{\hat{Y}}_{\text{train}}^{\left(i\right)}\;$ 16. ${\hat{Y}}_{\text{ensemble},\mathrm{val}}^{\left(t\right)}=\sum_{i=1}^{n}w_{i}^{\left(t\right)}.{\hat{Y}}_{\mathrm{val}}^{\left(i\right)}\;$ 17. ${\hat{Y}}_{\text{ensemble},\text{train}}^{\left(t\right)}=\sum_{i=1}^{n}w_{i}^{\left(t\right)}.{\hat{Y}}_{\text{test}}^{\left(i\right)}\;$ 18. Computing training and validation loss: 19. ${\text{loss}}_{\text{train}}^{\left(t\right)}=\text{Loss}\_\text{Function}\left(y_{\text{train}},{\hat{Y}}_{\text{ensemble},\text{train}}^{\left(t\right)}\right)$ 20. ${\text{loss}}_{\mathrm{val}}^{\left(t\right)}=\text{Loss}\_\text{Function}\left(y_{\mathrm{val}},{\hat{Y}}_{\text{ensemble},\mathrm{val}}^{\left(t\right)}\right)$ 21. Early Stopping: 22. If ${\text{loss}}_{\mathrm{val}}^{\left(t\right)}<\mathrm{bes}t_{\text{loss}}$ : 23. best_loss = ${\text{loss}}_{\mathrm{val}}^{\left(t\right)},$ best_weights = current_weight, Count = 0 24. Else: 25. count = count + 1 26. If count >= patience: Break 27. Compute gradient of loss w.r.t. weights: 28. $\nabla L=\frac{1}{N}.\left({\hat{Y}}_{\text{ensemble},\text{train}}^{\left(t\right)}-y_{\text{train}}\right).{{\hat{Y}}_{\text{train}}^{\left(t\right)}}^{T}$ 29. Update weighs using gradient descent: 30. current_weights = current_weights - α.∇L

Gradient descent is the key component of our proposed algorithm, which optimizes the ensemble model by minimizing the custom loss function. By penalizing inaccurate predictions more severely, particularly when the model is overconfident, the binary-cross-entropy-based loss function ( Equation 1) ensures that the model can handle classification tasks effectively.

$${\text{loss}}_{i}=\left(y_{\text{true},i}.\log\left(y_{\text{pred},i}+\varepsilon \right)+\left(1-y_{\text{true},i}\right).\log\left(1-y_{\text{pred},i}+\varepsilon \right)\right)$$ (1)

A small constant ε = 1 X 10 ^−10^ is introduced to ensure numerical stability in the logarithmic calculations when the prediction probability approached 0 or 1. Gradient descent helps to iteratively update the weights of the base models by calculating the direction of the steepest descent of the loss function. By minimizing the average loss, which is the average binary cross-entropy across all data points ( Equation 2), gradient descent allows the model to adjust its predictions in a way that maximizes accuracy while balancing the contributions of each base model in the ensemble.

$$\text{loss}=\frac{1}{N}\sum_{i=1}^{N}{\text{loss}}_{i}$$ (2)

This iterative procedure continues until the loss converges, thereby guaranteeing that the ensemble model is fine-tuned to its optimal performance. The effectiveness of the algorithm also strongly depends on factors such as the learning rate, early stopping (patience), and aggregation strategy via weighted averages. The learning rate is a critical factor in solving the gradient descent optimization to achieve a trade-off between overshooting and slow convergence. Early stopping stops training when the model performance starts to improve to prevent overfitting. The “patience” parameter control number of iteration algorithm to wait before stopping, hence used to prevent over-fitting. Similarly, while aggregating, we can simply use weighted averaging by weights that best increase the overall predictive power of the model in the ensemble.

### Evaluation matrix

In the context of imbalanced datasets, it is important to look beyond accuracy to comprehensively understand performance. Accuracy measures the correct predictions, but when there are different numbers of records for each class, it could lead to misleading conclusions. Precision centers around the percentage of the positive predictions made by the model that are correct, meaning that it determines how good the model is at not making false positives. Recall, or Sensitivity, measures how well the model identifies true positives among them and might be important when attempting to capture a minority class. The F1-score is the harmonic mean of precision and recall, avoiding either false positives or false negatives that dominate the evaluation. An ROC curve shows the trade-off between true-positive and false-positive rates at various thresholds, which aids in understanding the discriminative ability of a model. The AUC measures the capability of the model to distinguish between positive and negative classes by plotting a line on the ROC curve. Given the imbalanced nature of our datasets, these metrics are crucial for ensuring a more nuanced and reliable assessment of the model performance. The formulas to compute the performance matrices are mentioned in Table 3.

**Table 3.** Performance matrices and their mathematical notations.

Performance matric	Mathematical notation
Accuracy (Acc)	$\frac{1}{n}\sum_{i=1}^{n}1\left(y_{i}=\hat{y_{i}}\right)$
Precision (P)	$\frac{\sum_{i=1}^{n}1\left(y_{i}=1\;\Lambda \;\hat{y_{i}}=1\right)\;}{\sum_{i=1}^{n}1\left(\hat{y_{i}}=1\right)\;}$
Recall (R)	$\frac{\sum_{i=1}^{n}1\left(y_{i}=1\;\Lambda \;\hat{y_{i}}=1\right)\;}{\sum_{i=1}^{n}1\left(y_{i}=1\right)\;}$
F1-Score	2 x (P x R) / (P + R)
Area Under Curve (AUC)	$\mathrm{AUC}\approx \sum_{i=1}^{n-1}\left(\;{\mathrm{TPR}}_{i}+{\mathrm{TPR}}_{i+1}\right)\times \frac{\left({\mathrm{FPR}}_{i+1}-{\mathrm{FPR}}_{i}\right)}{2}$

Note: Where n is the total number of observations in the dataset and y _i_ and $\hat{y_{i}}$ are the true and false labels for the i ^th^ sample, respectively.

## Experimental results

The experimental results of our ensemble model are presented in this section, and an in-depth analysis of its performance over several datasets is carried out. The effectiveness of GBWOEM is evaluated by both the variant GBWOEM-R (random initialization) and GBWOEM-U (uniform initialization) on five datasets with various dimensionalities and characters. Given the diversity of base models used in the ensemble: LR, DT, KNN, RF, and MLP, we methodically assessed combinations of two, three, four, and all five base models. We quantified the quality of these results using standard metrics and drew particular attention to how different weight initialization strategies affect the generalizability of an ensemble across datasets with different dimensionalities.

### Breast cancer dataset

**Figure 3.**
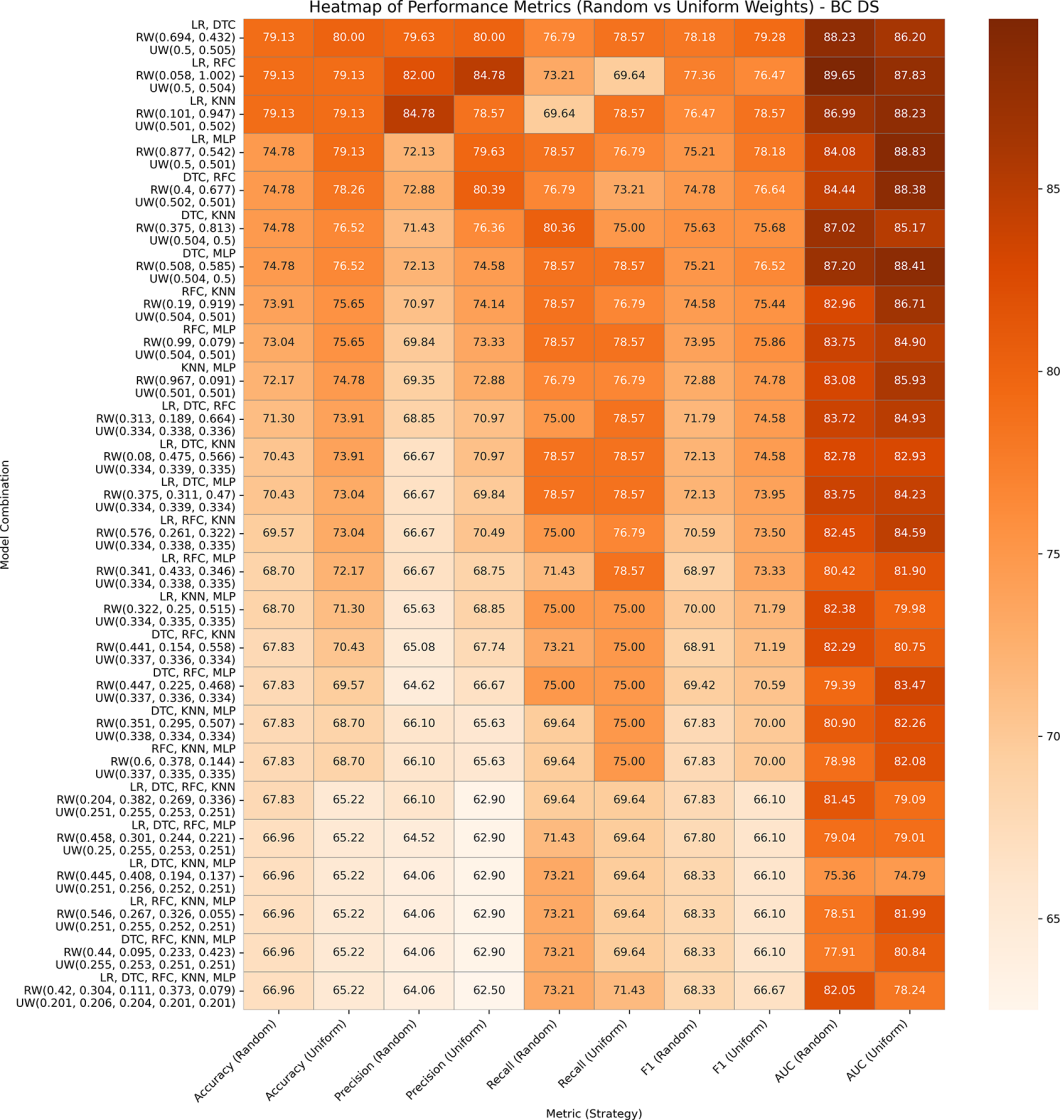
Performance matrices of GBWOEM (R&U) on Breast Cancer Dataset.

This dataset has 369 entries, which has 31 features altogether derived from FNA images of breast tumors, with diagnosis as the target variable. A performance analysis of both variants of GBWOEM is presented in Figure 3. In GBWOEM-R, the LR (0.058) and RFC (1.002) pairs achieve the highest test accuracy and AUC values, where RFC having a significantly higher weight, indicating its stronger contribution. In GBWOEM-U, although the LR (0.5) and DTC (0.505) pair gave the highest accuracy when we considered both accuracy and AUC, the LR (0.5) and MLP (0.501) pair performs best. For both pairs, the weights of the base models are almost equal, suggesting a more balanced contribution from each base model. Pairs such as (LR, DTC), (LR, RFC), and (LR, KNN) are performing consistently across both variants, underlining their robustness. However, in both the models, increasing the number of base models led to overfitting, increases the training accuracy to 100%, but reduction in the test accuracy. This highlights the importance of careful base model selection and weight optimization to avoid overfitting when scaling up the ensemble.

### Pima Indians diabetes database

**Figure 4.**
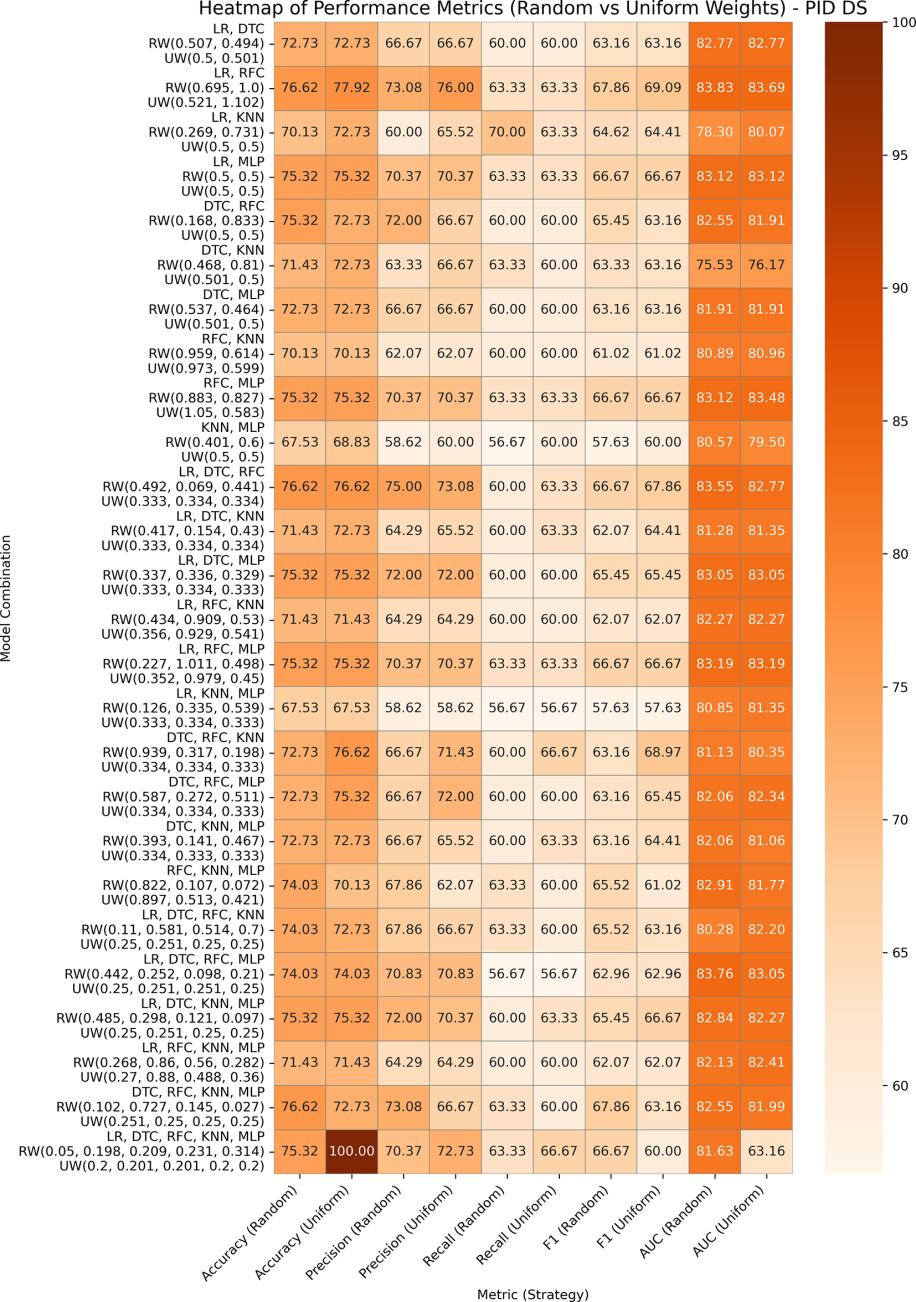
Performance matrices of GBWOEM (R&U) on Pima Indians Diabetes Database.

This is a common dataset utilized for research on diabetes and machine learning, which contains eight medical predictor variables, one target variable, and 768 entries. After data pre-processing, it is noticed that some columns have invalid 0’s, which are replaced with the min or median of those columns. The performances of both variants of our proposed ensemble model are presented in Figure 4. In GBWOEN-R, the (LR, RFC) (0.695, 1.0) and (DTC, RFC, KNN, MLP) (0.102, 0.727, 0.145, 0.027) pairs showed the highest accuracy, but (LR, RFC) achieved a higher AUC with RFC having the highest weights and more contribution to the final ensemble result. In GBWOEM-U, the combination of all five base models yielded the highest accuracy but a low AUC value. Again, (LR, RFC) (0.521, 1.102) achieves a balanced accuracy and AUC, with RFC dominating.

### Diabetic Retinopathy Debrecen Database

**Figure 5.**
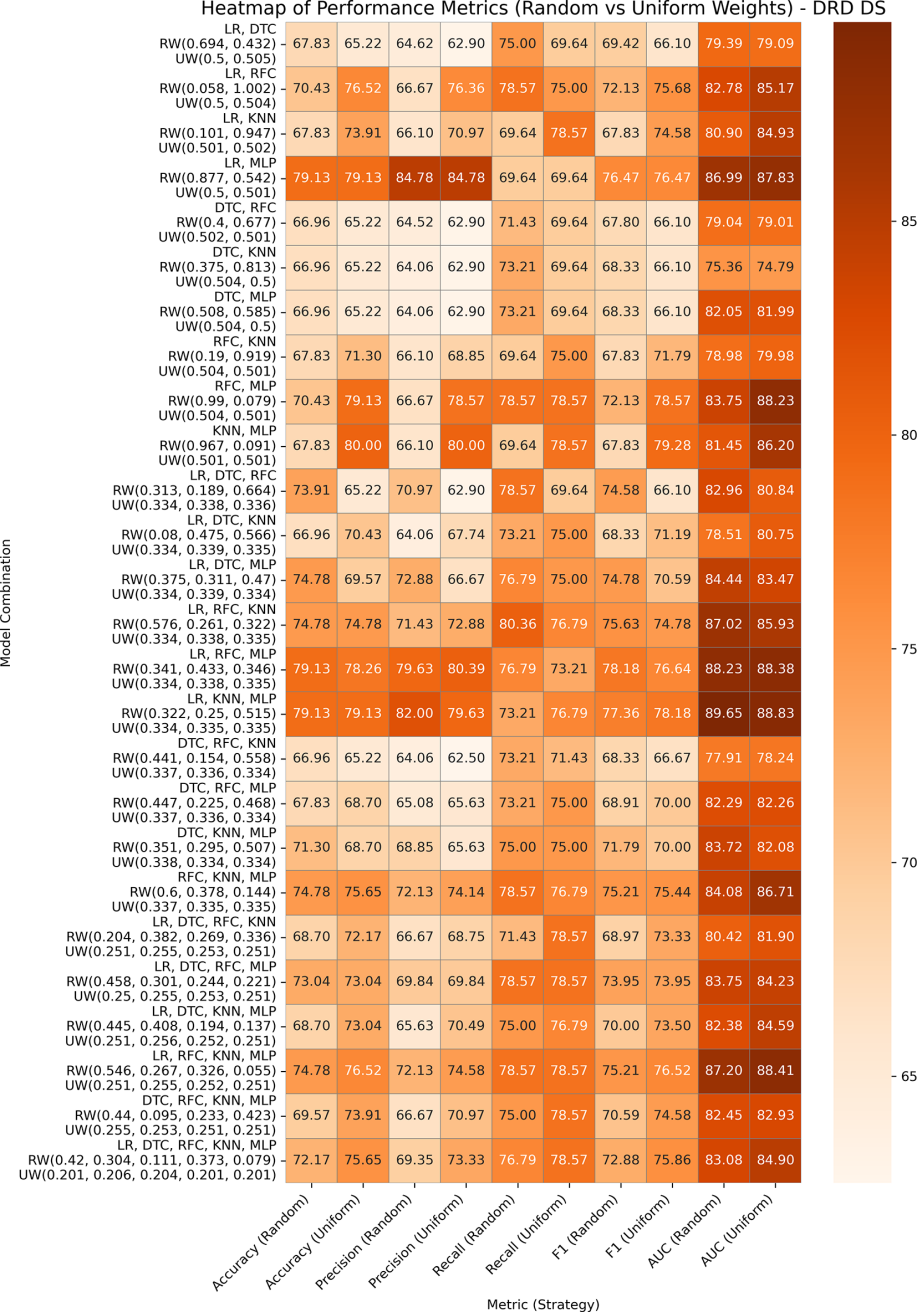
Performance matrices of GBWOEM (R&U) on Diabetic Retinopathy Database.

The Diabetic Retinopathy Debrecen Database, comprising 1,151 instances and 19 features, helps predict diabetic retinopathy using image-driven features. The performances of GBWOEM-R and GBWOEM-U are represented in Figure 5. In GBWOEM-R, (LR, MLP) (0.877, 0.542), (LR, RFC, MLP) (0.341, 0.433, 0.346), and (LR, KNN, MLP) (0.322, 0.25, 0.515) achieved the highest accuracy, whereas (LR, KNN, MLP) had the best AUC. From the final weight of each base model, it can be concluded that all models contribute significantly to the final result. The GBWOEM-U variant achieved slightly higher accuracy than GBWOEM-R, but had a lower AUC value. In this variant, (KNN, MLP) (0.501, 0.501) showed higher accuracy, where each model contributed equally. However, as per the AUC concern, the (LR, KNN, MLP) pair provides balanced results for both variants.

### Obesity database

**Figure 6.**
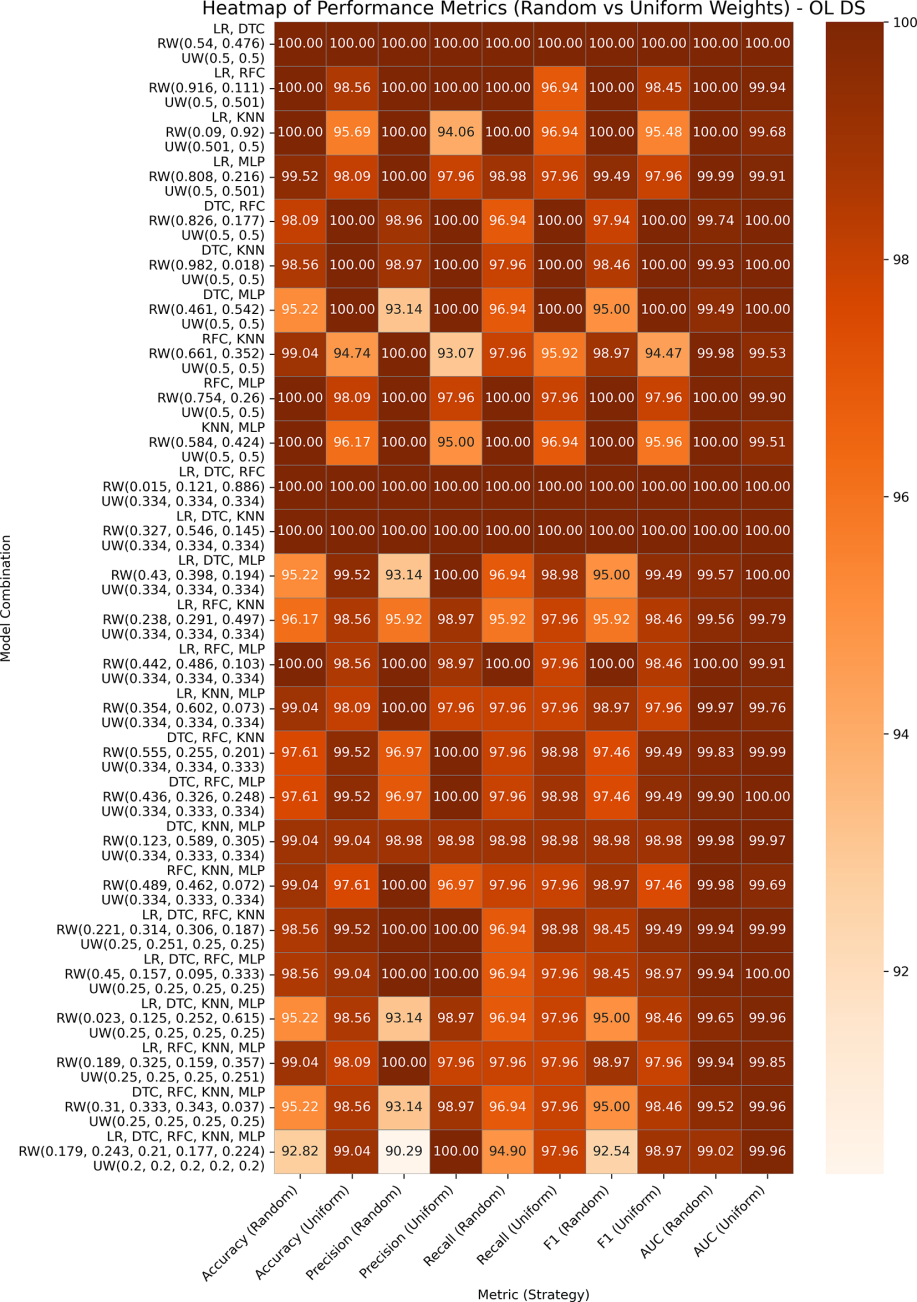
Performance matrices of GBWOEM (R&U) on Obesity Database.

This dataset includes 2,111 instances and 17 features related to lifestyle and dietary habits. The target column is divided into different classes, including normal weight, underweight, overweight, and obese (I, II, and III). For our experiment, we reclassified the target variable into binary classes, classifying all obesity levels as class 1 and other classes as 0 because the goal is to detect obesity. The performance analyses of GBWOEM-R and GBWOEM-U on this dataset are represented in Figure 6.

Both variants performed exceptionally well, with accuracy and AUC values reaching or exceeding 99% across all combinations. In all GBWOEM-U pairs, weights are evenly distributed with minimal adjustment. Pair-like (LR, DTC), (LR, KNN), and (LR, RFC) algorithms continue to perform well, consistent with their performance across other datasets. For this dataset, the higher-order combinations (e.g., 5C3-5C5) perform well in the testing phase, and the training-testing accuracy gap is minimal, suggesting better generalization, possibly due to the nature of the attributes or the binary classification approach we employed.

### Thyroid disease

**Figure 7.**
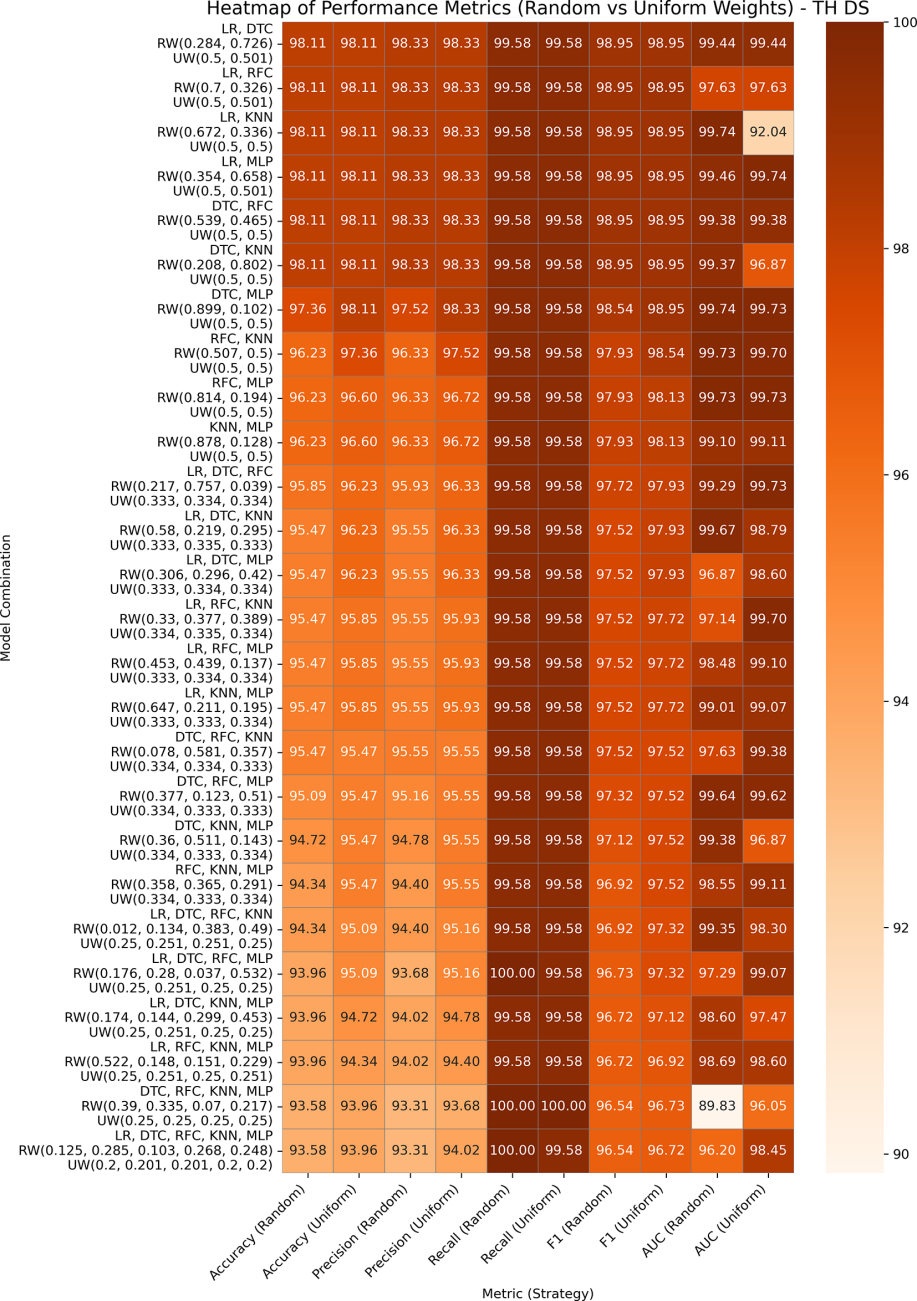
Performance matrices of GBWOEM (R&U) on Thyroid Disease.

Hypothyroid Disease includes 3,711 entries and 30 features for the detection of hypothyroidism. Both the variants GBWOEM-R and GBOWEM-U are performing well, where almost all the pairs are achieving accuracy over 93% and AUC over 96%, as presented in Figure 7. Similar to the Obesity dataset, the difference between the training and testing results is minimal, and higher-order combinations also perform well, likely resulting in a large sample size in both datasets. In GBWOEM-R, models such as DTC and RFC contribute more to their corresponding pairs with respect to other models. In GBWOEM-U, almost all base models have equal weights in their respective pairs and require minimal weight correction during optimization. This balance suggests a uniform contribution from all models, which may contribute to strong performance across multiple metrics.

### Comparison with existing ensemble model

This section of the Results and Discussion section shows a comparison between GBWOEM and other existing ensemble models: Adaboost, Catboost, GradientBoost, LightGBM, and XGBoost. This will help us establish the potential benefits of our method in terms of precision, AUC, and generalization capability. Our model led to the enhancement of test accuracy compared to existing ensemble models, with improvements between 0.48% and 8.26% across all five datasets. The specific accuracy gains for each dataset were as follows: Breast Cancer (5.32%), Pima Indians Diabetes Database (2.60%), Diabetic Retinopathy Debrecen (8.26%), Obesity Level estimation based on physical condition and eating habits (0.48%), and Thyroid Disease (2.32%). Although these values differ in the amount of improvement they contribute, this highlights that one of the great features of GBWOEM is that it is capable of handling various datasets and tasks efficiently.

**Figure 8.**
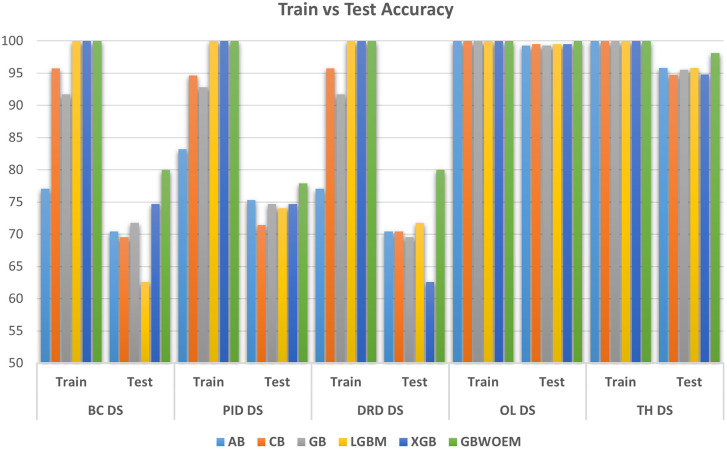
Comparison of training and testing accuracy between existing and proposed ensemble model for all the datasets.

**Figure 9.**
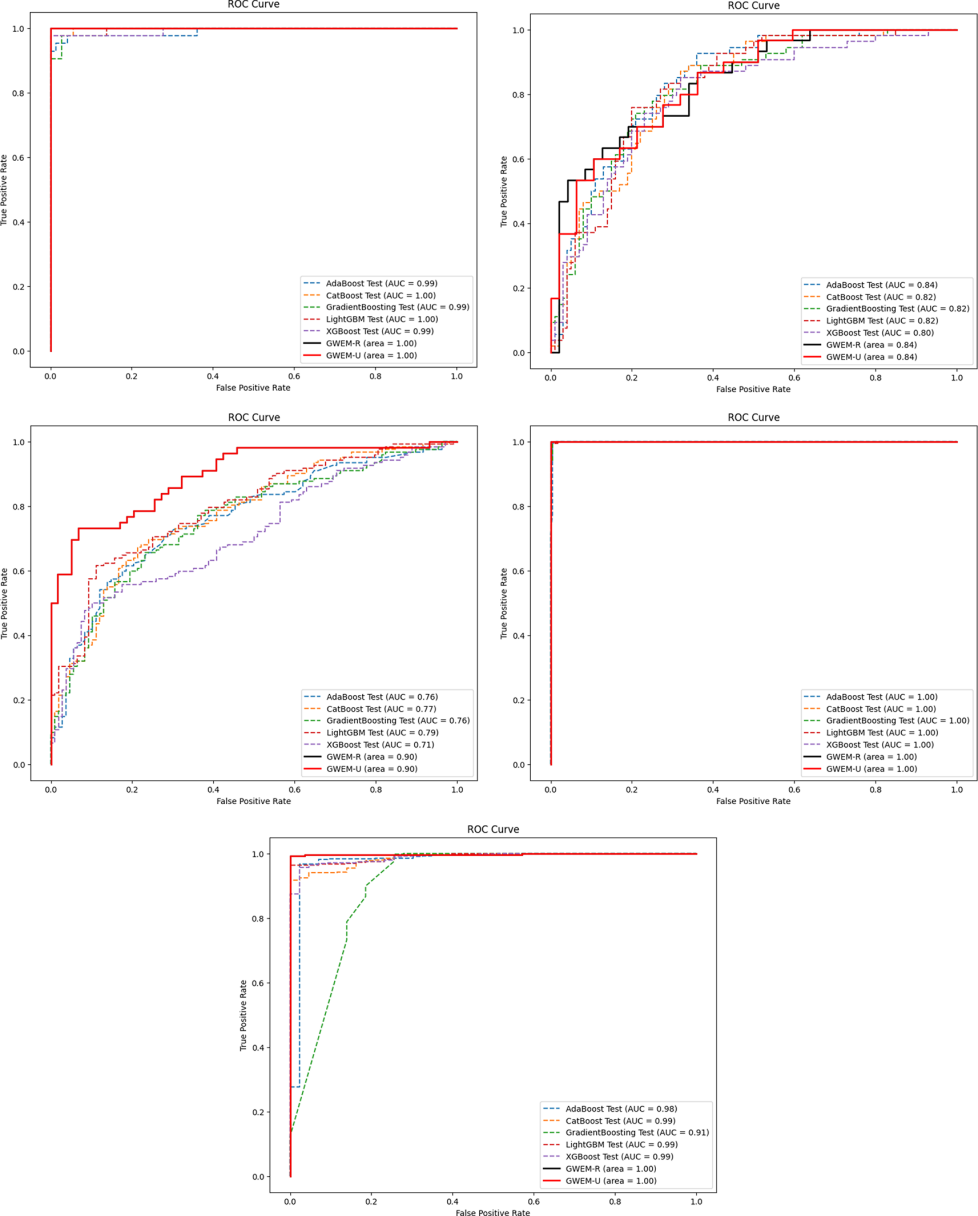
ROC Plot of existing and proposed GBWOEM (R&U) model (a) BC DS, (b) PID DS, (c) DRD DS, (d) OL DS and (e) TH DS.

A comprehensive comparative quantitative performance for the details is given in Table 4 between the training and test accuracies of both the GBWOEM and existing models. In addition, ROC curves were drawn for each dataset for these five ensemble models and two variants of GBWOEM (GBWOEM-R and GBWOEM-U). It is very useful in the case of imbalanced datasets and helps us better understand how the model separates classes. From Figure 9, we can see that both variants of the proposed GBWOEM are able to distinguish between classes well for all datasets except PID DS. The models shows comparable results (80-90%) for performance matrices like precision, recall and F1-score, which rules out overfitting and is evident in Figures 3, 6, and 7. A bar chart ( Figure 8) is also included to show the training and test accuracies of the baseline models vs. our additionally proposed GBWOEM variants to graphically display these differences in our model accuracy.

**Table 4.** Test accuracy and test AUC comparison between GBWOEM and existing ensemble models.

Dataset	Test accuracy						Test AUC					
	AB	CB	GB	LGBM	XGB	GBWOEM	AB	CB	GB	LGBM	XGB	GBWOEM
BC DS	70.43	69.57	71.74	62.61	74.68	80	70.38	69.98	72.14	61.41	69.85	89.65
PID DS	75.32	71.43	74.68	74.03	74.68	77.92	71.2	67.35	70.28	69.78	69.85	83.69
DRD DS	70.43	70.43	69.57	71.74	62.61	80	71.12	70.38	69.98	72.14	61.41	89.65
OL DS	99.28	99.52	99.28	99.52	99.52	100	99.26	99.49	99.26	99.49	99.49	100
TH DS	95.79	94.73	95.54	95.79	94.79	98.11	82.35	87.11	85.94	82.35	84.47	99.74

## Conclusion and future work

In this study, we proposed the Gradient-Based Weight Optimized Ensemble Model (GBWOEM) consisting two variants, GBWOEM-R (random initialization) and GBWOEM-U (uniform initialization), designed to improve classification performance by dynamic weight optimisation for LR, DTC, KNN, RFC and MLP base models. Using the weighted average approach, the model’s weights are treated as real-valued variables optimized using gradient descent. The model was evaluated on five diverse datasets: Breast Cancer, Pima Indians Diabetes Database, Diabetic Retinopathy Debrecen, Obesity Level estimation based on physical condition and eating habits, and Thyroid Disease, each with unique characteristics and dimensions. In the end, we observed significant improvements in test accuracy across all datasets, with a gain of 0.48% to 8.26%, as compared to existing ensemble models, namely Adaboost, Catboost, GradientBoost, LightGBM, and XGBoost. GBWOEM achieved its highest increase in accuracy on the Diabetic Retinopathy Debrecen dataset, suggesting that GBOWOEM effectively addresses complex, feature-rich datasets. While both GBWOEM variants showed similar functional behaviour, GBWOEM-R favoured certain based models like RFC due to uneven weight distribution, but GBWOEM-U showed even distribution of weights and delivered more balanced and stable results across the dataset. In addition, the ROC curves and AUC values confirmed GBWOEM’s robustness of GBWOEM on various datasets. Surprisingly, increasing the number of base models contextually increases the training accuracy (up 100% in some cases) but not test performance, emphasizing the risk of overfitting in ensemble models. The dynamic weight optimization of the GBWOEM was shown to be a key strength, allowing flexibility across datasets with different dimensions and class distributions. Future work aims to incorporate advanced weight optimization techniques, such as adaptive learning rates or metaheuristic approaches, and test the model in multi-class and large-scale datasets for broader validation while maintaining lower computational complexity.

## Data Availability

The datasets used in this research are publicly available and can be accessed through the following DOIs:
• Diabetic Retinopathy Debrecen ( https://archive.ics.uci.edu/dataset/329/diabetic+retinopathy+debrecen; doi:10.24432/C5XP4P), • Estimation of Obesity Levels Based On Eating Habits and Physical Condition ( https://archive.ics.uci.edu/dataset/544/estimation+of+obesity+levels+based+on+eating+habits+and+physical+condition; doi:10.24432/C5H31Z), • Thyroid Disease ( https://archive.ics.uci.edu/dataset/102/thyroid+disease; doi:10.24432/C5D010). • The Pima Indians Diabetes Dataset, originally hosted on UCI ML Repository is no longer available there. However, it can be accessed via Kaggle at https://www.kaggle.com/datasets/uciml/pima-indians-diabetes-database .
