# Supplementary material for: GBWOEM: A Gradient-Based Weight Optimization Model for Improved Predictive Accuracy in Healthcare
Source: F1000Res. 2026 Jan 27;14:1161. Originally published 2025 Oct 24. [Version 2] doi: 10.12688/f1000research.169436.2 (PMC12906645; doi:10.12688/f1000research.169436.2)
Supplement: Peer review history

# Reviewer report: Reviewer response for version 2

**DOI:** 10.5256/f1000research.195371.r458348

**Published:** 2026-02-14

**Authors:** Anjum John

**Affiliations:** 1 Pushpagiri Medical College Hospital, Thiruvalla, Kerala, India

Thank you for allowing me to review this paper which is technically sound and is indexable material. There is novelty and a domain of technical contribution, with acceptable experimental benchmarking but  there are a few concerns about its theoretical depth, clinical translation, and external validation.

**Major Strengths**

  1. Novel Methodological Idea: The core idea — gradient-based dynamic weight optimization across heterogeneous base models — is  relevant. The model’s combination of LR, DT, RF, MLP, KNN uses gradient descent to optimize ensemble weights dynamically and shows measurable performance gain (0.48–8.26%) vs standard ensembles. This novelty in applied ML is indexable though theoretical novelty may be insufficient.

 2. Multi-Dataset Evaluation: The model used five healthcare datasets across domains were used — improves perceived generalizability.

 3. Proper ML Pipeline Elements used included Grid search CV, Train/validation/test splits, Early stopping, and Multiple metrics (AUC, F1, precision, recall)- which meets baseline reproducibility expectations.

 4. Transparency Elements: Because public datasets used are listed, and the algorithm pseudo code provided, there is an element of transparency.

**Major Concern**

**1. Weak Theoretical Foundation:** Though the model uses gradient descent, evidence of convergence behaviour, Stability conditions, Complexity scaling and Sensitivity to hyperparameters are not explained in detail. This has been flagged by other reviewers in terms of the lack of detailed theoretical rationale for the use of this model. Clarity on the theoretical model is necessary to improve the depth of understanding of the topic as a new one. Suggestions are to add, discussion on convexity, empirical convergence plots, and sensitivity analyses like learning rates, patience, and finalization.

 2. **Interpretability **— As it stands the manuscript shows weight values without clinical meaning being demonstrated. Previous reviews have stated that the study focuses on accuracy but lacks interpretability; add SHAP/LIME. Healthcare ML publication require transparency in contributions, clinician usability, and decision pathway explanations. Please provide at least one of the following: SHAP global + local explanations, feature importance stability across folds, or case-based explanation examples. 

 3. **External Validation Missing: **For clinical MI credibility after publication, at least one of external dataset testing, Nested CV, or leave-one-dataset-out validation might be performed.

 4. **Possible Overfitting Signals**: The authors show 100% training accuracy in some models with test performance dropping when model complexity increases. The authors can provide learning curves, calibration plots, or bootstrapped CI for AUC.

 5. Class Imbalance Handling Not Methodologically Strong because the authors rely mostly rely on natural distribution, avoid SMOTE, and argue that performance metrics are stable. Not provided are PR-AUC reporting, Cost-sensitive learning, or Threshold optimization

 6. **Clinical Relevance Is Mostly Theoretical: **The paper claims that this method Improves patient outcomes, with diagnostic consistency but there was no clinical deployment scenario, and no decision threshold analysis.

 7. **Dataset Choice Is Safe but Not Challenging:** The datasets used for the stuffy are good for method comparison but weak for real clinical impact assessment.

 8. Computational Costs important for regular use of the model like training time, memory, and scalability to large HER datasets are not assessed.

 9. The inconsistencies in grammar, repetition of texts, density of figures, and claims of larger uses than that shown by the data, are minor flaws in the manuscript.

**Recommendations**: The manuscript presents a technically sound and novel ensemble optimization approach with consistent improvements across benchmark healthcare datasets. However, before strong endorsement, the work would benefit from enhanced theoretical grounding of the optimization procedure, additional validation on external datasets, and improved interpretability analyses to support real-world clinical applicability.

Is the work clearly and accurately presented and does it cite the current literature?

Yes

If applicable, is the statistical analysis and its interpretation appropriate?

Yes

Are all the source data underlying the results available to ensure full reproducibility?

Yes

Is the study design appropriate and is the work technically sound?

Partly

Are the conclusions drawn adequately supported by the results?

Yes

Are sufficient details of methods and analysis provided to allow replication by others?

Partly

Reviewer Expertise:

Qualitative Research Methods, Medical Students, Teaching, Learning, Public health

I confirm that I have read this submission and believe that I have an appropriate level of expertise to confirm that it is of an acceptable scientific standard, however I have significant reservations, as outlined above.

# Reviewer report: Reviewer response for version 1

**DOI:** 10.5256/f1000research.186779.r442725

**Published:** 2026-01-07

**Authors:** Helen D

**Affiliations:** 1 SRM Institute of Science and Technology, Kattankulathur, Tamil Nadu, India

1. **Novelty and Contribution:**
  The development of GBWOEM shows the meaningful advancement in ensemble learning method, with a clear focus on weight optimization. The proposed model is compared with standard algorithms and achieves improved accuracy, which highlights its potential contribution.
2. **Methodology:**
  The implementation of two different weight initialization methods—random (GBWOEM-R) and uniform (GBWOEM-U)—and the comparative analysis effectively show the impact of weight initialization on ensemble performance.
3. **Interpretability:**
  The paper could benefit from a more detailed explanation of how the optimized weights of base models contribute to the final predictions. This would enhance the interpretability and practical relevance of the model, especially in healthcare applications.
4. **Class Imbalance:**
  The datasets used are reported to have class imbalance. The authors should clarify how this imbalance was addressed during training and evaluation to ensure the results are reliable.
5. **Overfitting Concern:**
  In Figure 9, ROC curves with AUC values near 1.00 may indicate potential overfitting. The authors should verify these results, possibly by evaluating the models on independent or cross-validation datasets to ensure generalizability.

Is the work clearly and accurately presented and does it cite the current literature?

Partly

If applicable, is the statistical analysis and its interpretation appropriate?

Partly

Are all the source data underlying the results available to ensure full reproducibility?

Partly

Is the study design appropriate and is the work technically sound?

Yes

Are the conclusions drawn adequately supported by the results?

Partly

Are sufficient details of methods and analysis provided to allow replication by others?

Partly

Reviewer Expertise:

Machine Learning, Deep Learning

I confirm that I have read this submission and believe that I have an appropriate level of expertise to confirm that it is of an acceptable scientific standard, however I have significant reservations, as outlined above.

## References

[1] : Big data-driven optimal weighted fused features-based ensemble learning classifier for thyroid prediction with heuristic algorithm. *Journal of Combinatorial Optimization* .2025;49(4) : 10.1007/s10878-025-01304-4 10.1007/s10878-025-01304-4

# Response

**Published:** 2026-01-16

**Authors:** Satyananda Champati Rai

**Affiliations:** School of Computer Engineering, Kalinga Institute of Industrial Technology, Bhubaneswar, Odisha, India

**Q1. The paper could benefit from a more detailed explanation of how the optimized weights of base models contribute to the final predictions. This would enhance the interpretability and practical relevance of the model, especially in healthcare applications.**

**Response:**

 We thank the reviewer for this valuable suggestion regarding interpretability. To address this, we have expanded the result to explicitly detail how the optimized weights reflect the contribution of each base model.

 For example, as noted in the revised manuscript, in the GBWOEM-R variant, the Random Forest Classifier (RFC) was assigned a significantly higher weight (1.002) compared to Logistic Regression (0.058), quantitatively demonstrating RFC's stronger contribution to the decision boundary for this specific dataset. Additionally, as requested, we have updated the relevant performance comparison figures to include the final weights of Random Weight (RW) and Uniform Weight (UW) initialization strategies. This visual comparison highlights how our optimization method specifically identifies and prioritizes the most reliable classifiers for healthcare diagnostics, rather than relying on static or arbitrary averaging.

**Q2. The datasets used are reported to have class imbalance. The authors should clarify how this imbalance was addressed during training and evaluation to ensure the results are reliable.**

**Response:**

 We appreciate the reviewer highlighting this important aspect of data validity. We have analysed the class distribution for all five datasets used in the study. Four of the datasets exhibit a ratio approximating 60:40, which falls within the range of practical balance where standard classifiers perform effectively without intervention.

 For the dataset exhibiting an imbalance, we empirically evaluated the model's performance without synthetic balancing (such as SMOTE). We have clarified in the Methodology section that a part of the dataset is kept as test data set to test the model performance in unseen data. We observed that the model converged successfully with a negligible gap between training and testing performance metrics (specifically, Recall and F1-score). This indicates that the feature space was sufficiently distinct to allow the model to learn the minority class boundaries effectively. We have clarified that we avoided synthetic oversampling for this specific case to prevent the introduction of noise and to ensure the evaluation reflects the model's robustness on naturally occurring data distributions.

**Q3. In Figure 9, ROC curves with AUC values near 1.00 may indicate potential overfitting. The authors should verify these results, possibly by evaluating the models on independent or cross-validation datasets to ensure generalizability.**

**Response:**

 We agree that AUC values approaching 1.00 warrant careful scrutiny. We have re-verified the results presented in Figure 9. The high AUC values are attributed to the high separability of the datasets, which is consistent with existing literature on the respective datasets.

 Due to strict page length constraints in the submitted manuscript, we could not include the exhaustive Train-vs-Test comparison data for every model variant. However, to address the reviewer’s concern, we have provided this detailed analysis below (see Table 1).  As illustrated in Table 1, the performance metrics (especially F1-score, recal,l and precision) for both training and testing phases fall within a consistent range with minimal deviation. The absence of a significant drop in performance from Training to Testing confirms that the model generalizes well to unseen data and is not memorizing the training set. We have added a statement to the Results section clarifying that this stability across splits validates the high AUC scores.

**Table 1:** Performance matrices of GBWOEM-R on Breast Cancer Dataset (up to 3 model pairs)

https://docs.google.com/spreadsheets/d/1BFQRoqDtJme5ZrmqXKYFwuNuKaGeyq7vQyu6L3k_PYo/edit?usp=sharing

# Reviewer report: Reviewer response for version 1

**DOI:** 10.5256/f1000research.186779.r427169

**Published:** 2025-11-20

**Authors:** HEMA PRIYA K

**Affiliations:** 1 Easwari Engineering College, Chennai, Tamil Nadu, India

This manuscript presents a novel ensemble learning framework — Gradient-Based Weight Optimized Ensemble Model (GBWOEM)—which introduces a gradient-based weight optimization strategy for improving predictive accuracy in healthcare classification tasks. Two variants (GBWOEM-R and GBWOEM-U) are developed and evaluated across five diverse healthcare datasets.

 The model is compared with well-established ensemble methods such as AdaBoost, CatBoost, GradientBoost, LightGBM, and XGBoost. The study is technically sound and methodologically comprehensive, offering meaningful improvements in prediction accuracy.

 However, certain aspects require deeper theoretical justification, statistical validation, and

 refinement to enhance its scientific robustness, clarity, and clinical relevance.

**Observation 1:**The paper outlines the use of gradient descent for weight optimization but lacks a detailed theoretical rationale for convergence, stability, or hyperparameter selection.

**Recommendation 1:**Include a mathematical discussion or citation to establish convergence guarantees, sensitivity analysis, and computationalcomplexity details.

**Observation 2:** The study focuses on accuracy but lacks interpretability, crucial for healthcare AI.

**Recommendation 2: ** Add analyses using SHAP, LIME, or feature importance to show clinical relevance.

 The manuscript demonstrates strong potential and novelty but requires theoretical, statistical, and interpretive enhancements. Addressing these revisions will significantly improve its scholarly rigor and readiness for indexing.

Is the work clearly and accurately presented and does it cite the current literature?

Yes

If applicable, is the statistical analysis and its interpretation appropriate?

Partly

Are all the source data underlying the results available to ensure full reproducibility?

Yes

Is the study design appropriate and is the work technically sound?

Yes

Are the conclusions drawn adequately supported by the results?

Yes

Are sufficient details of methods and analysis provided to allow replication by others?

Yes

Reviewer Expertise:

Thyroid Disorders, Deep Learning, Machine Learning, RNN, DNN, CNN, AI

I confirm that I have read this submission and believe that I have an appropriate level of expertise to confirm that it is of an acceptable scientific standard.

## References

[1] : Intelligent Fusion of Heuristically Optimized 1DCNN with Weighted Optimized DNN for Thyroid Disorder Prediction Framework. *International Journal of Information Technology & Decision Making* .2025;24(05) : 10.1142/S0219622025500105 1397-1433 10.1142/S0219622025500105

[2] : Big data-driven optimal weighted fused features-based ensemble learning classifier for thyroid prediction with heuristic algorithm. *Journal of Combinatorial Optimization* .2025;49(4) : 10.1007/s10878-025-01304-4 10.1007/s10878-025-01304-4

[3] : Innovative Framework for Thyroid Disease Detection by Leveraging Hybrid AGTEO Feature Selection and GRU Classification Model. *International Research Journal of Multidisciplinary Technovation* .2024; 10.54392/irjmt2439 112-127 10.54392/irjmt2439
